# Supplementary material for: Cell geometry regulates tissue fracture
Source: Nat Commun. 2023 Dec 13;14:8275. doi: 10.1038/s41467-023-44075-4 (PMC10719271; doi:10.1038/s41467-023-44075-4)
Supplement: Supplementary file 1 — Supplementary Information [file 41467_2023_44075_MOESM1_ESM.pdf]

Supplementary Information

Cell Geometry Regulates Tissue Fracture

Amir J. Bidhendi[1,3,*], Olivier Lampron[2], Frédérick P. Gosselin[2], Anja Geitmann[1,*]

[1]Department of Plant Science, McGill University, Macdonald Campus, 21111 Lakeshore, Ste-Anne-de-Bellevue, Québec, H9X 3V9, Canada.

[2]Laboratoire de Mécanique Multi-échelles, Département de génie mécanique, École Polytechnique de Montréal, Montreal, Québec, H3C 3A7, Canada.

*Corresponding author. Email: amir@bidhendi.net, geitmann.aes@mcgill.ca

[3]Current affiliation: EERS Global Technologies, Montreal, Canada.

This file includes:

Supplementary Note 1
Supplementary Figs. 1 to 4

Supplementary Note 1.

Supplementary Note 1.1. Tear test of notched specimens

The edge-notched specimen occasionally warped, which complicated real-time observation of tear progression due to the sample's lack of flexural rigidity. Center-notched sample geometry was adopted to address this issue. These loading and sample geometry conditions were seen to mitigate the issues with the edge-notched test keeping the specimen relatively flat and allowing optical microscopy. Because of the geometry of the center-notched samples, the propagating tear is prevented from following the path of least resistance, but it is forced to traverse the width of the sample. As a result, this test is better suited to quantifying the tear resistance of different samples.

Supplementary Note 1.2. Failure of plant tissue under tension

In this study, we first investigated the extent to which the failure of an intact tissue under tensile load occurs due to cell-cell detachment in the tissue. We hypothesized that tissues with wavy cell shapes should have a higher tensile strength because wavy cells have increased contact areas and are harder to detach from one another. To investigate this, strips of leaves from wild-type and *any1 Arabidopsis* were examined in tension-to-failure tests. The mutant *any1* possesses an altered cellulose crystallinity with pavement cells that appear swollen and less wavy compared to the wild type[1]. Samples that failed in the gauge area were immediately chemically fixed and observed under the scanning electron microscope. The observations indicated that, in both wild-type and *any1* sample rupture behavior was dictated primarily by the loading geometry: The rupture tends to cross the sample width following the shortest path combining both border and cell fractures. Therefore, we determined that due to the loading geometry and narrow width of the specimens, the effect of cell waviness on the tensile strength of samples cannot be accurately determined with this method. Additionally, we noted significant curvature of the fracture surfaces out of the xy plane, likely due to large strains before rupture as well as deformations occurring during the fixation and dehydration processes. This curvature obscures the free fracture edges and renders the precise observation of the tear path challenging (Supplementary Fig. 3C). However, away from the main ruptured edges, we observed that in both wild-type and *any1,* cracks can propagate into cell borders (Fig. 6E). Because of artifacts induced in fresh sample specimens, we chose to perform further fracture tests on dehydrated leaf specimens.

Reference

1. Fujita, M. *et al.* The anisotropy1 D604N mutation in the Arabidopsis Cellulose Synthase1 catalytic domain reduces cell wall crystallinity and the velocity of cellulose synthase complexes. *Plant Physiol.* **162**, 74–85 (2013).

Supplementary Figures

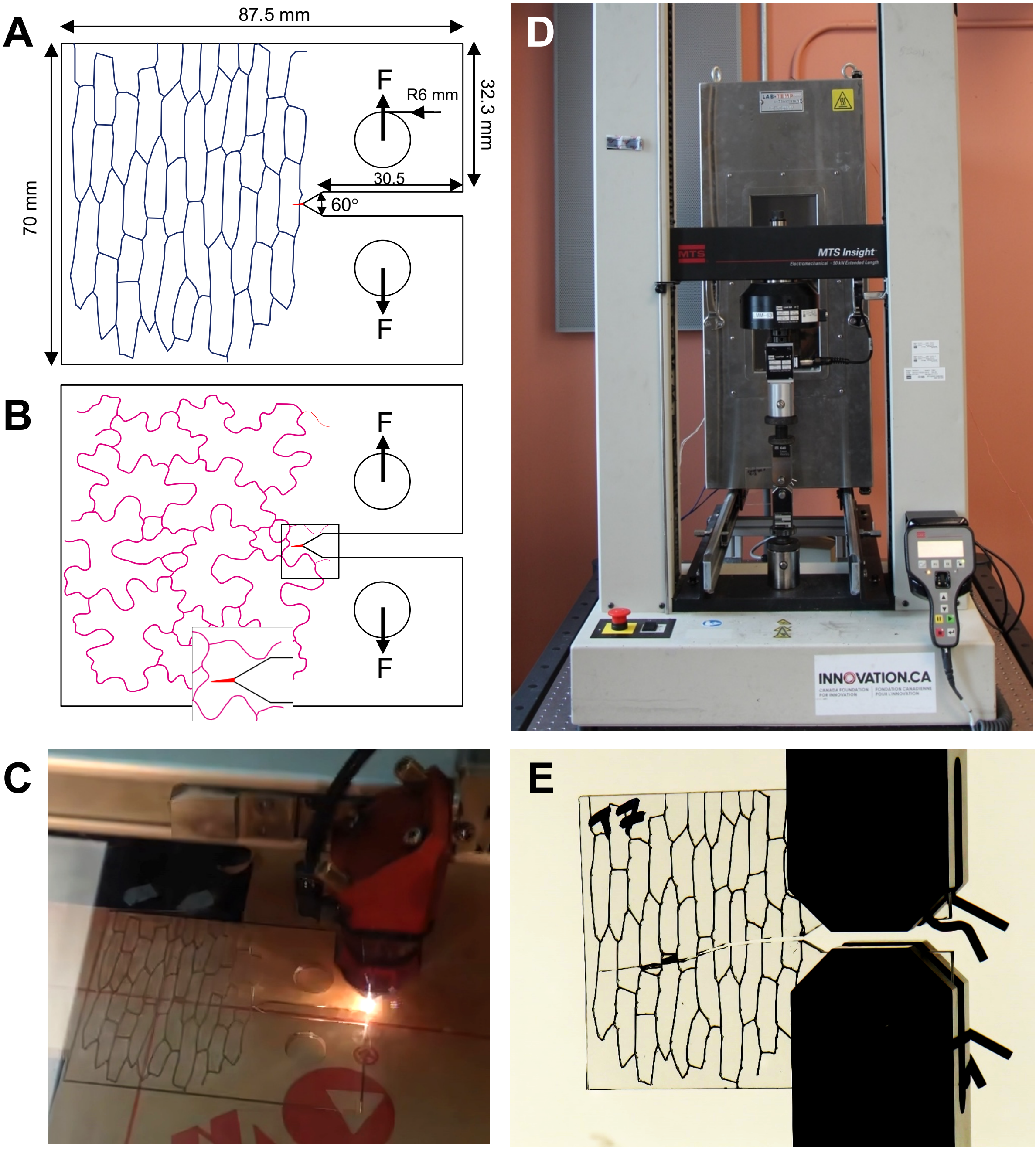

Supplementary Fig. 1. Fracture of laser engraved PMMA samples. Outlines of tissue with **A)** Brick-shaped (onion) cell and **B)** wavy *Arabidopsis* epidermal cell pattern extracted from microscopic images. The inset in (B) shows schematic of a microcrack (red triangle in A and B) that was induced at the tip of the cutout notch by a gentle tap of a blade. **C)** Vectorized patterns were laser engraved on cast PMMA and compact tension (CT) specimens were cut out for macroscale fracture tests. A total of 5 specimen types including control (no engraving) were prepared. **D)** PMMA fracture tests were performed on a MTS Insight mechanical testing device. **E)** Close-up view of the fractured CT specimen with brick-shaped cells aligned transversely to the initial crack orientation.

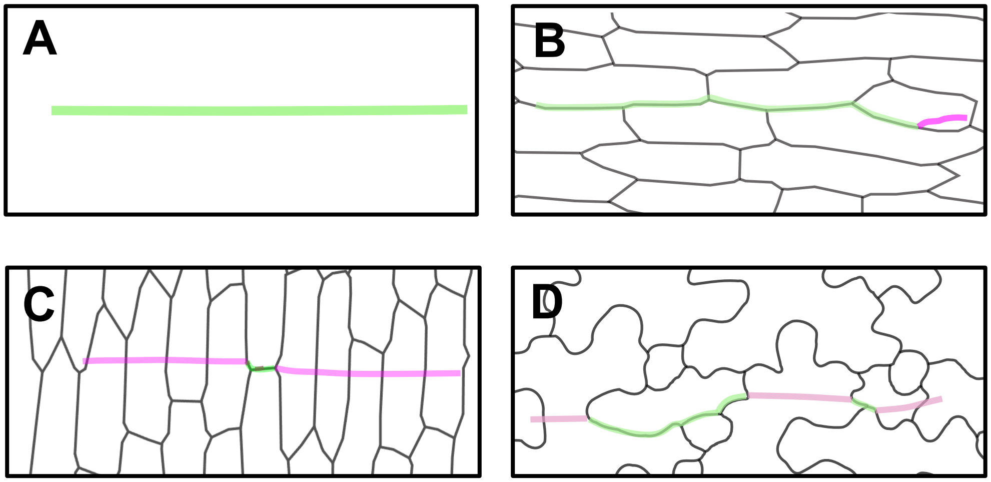

Supplementary Fig. 2. PFM simulation of crack propagation. Fracture behavior observed with the PFM for $\boldsymbol{E^{int}} = \boldsymbol{E^{cell}}$ and $\boldsymbol{G_c^{int}} = \boldsymbol{G_c^{cell}/2}$. Crack path obtained respectively for the **A)** Control, **B)** Brick-shaped onion epidermal cells aligned longitudinally, and **C)** Transversely to crack as well **D)** Wavy Arabidopsis cell patterns. The cell interfaces are outlined in black. See also Fig. 3 and Supplementary Fig. 4.

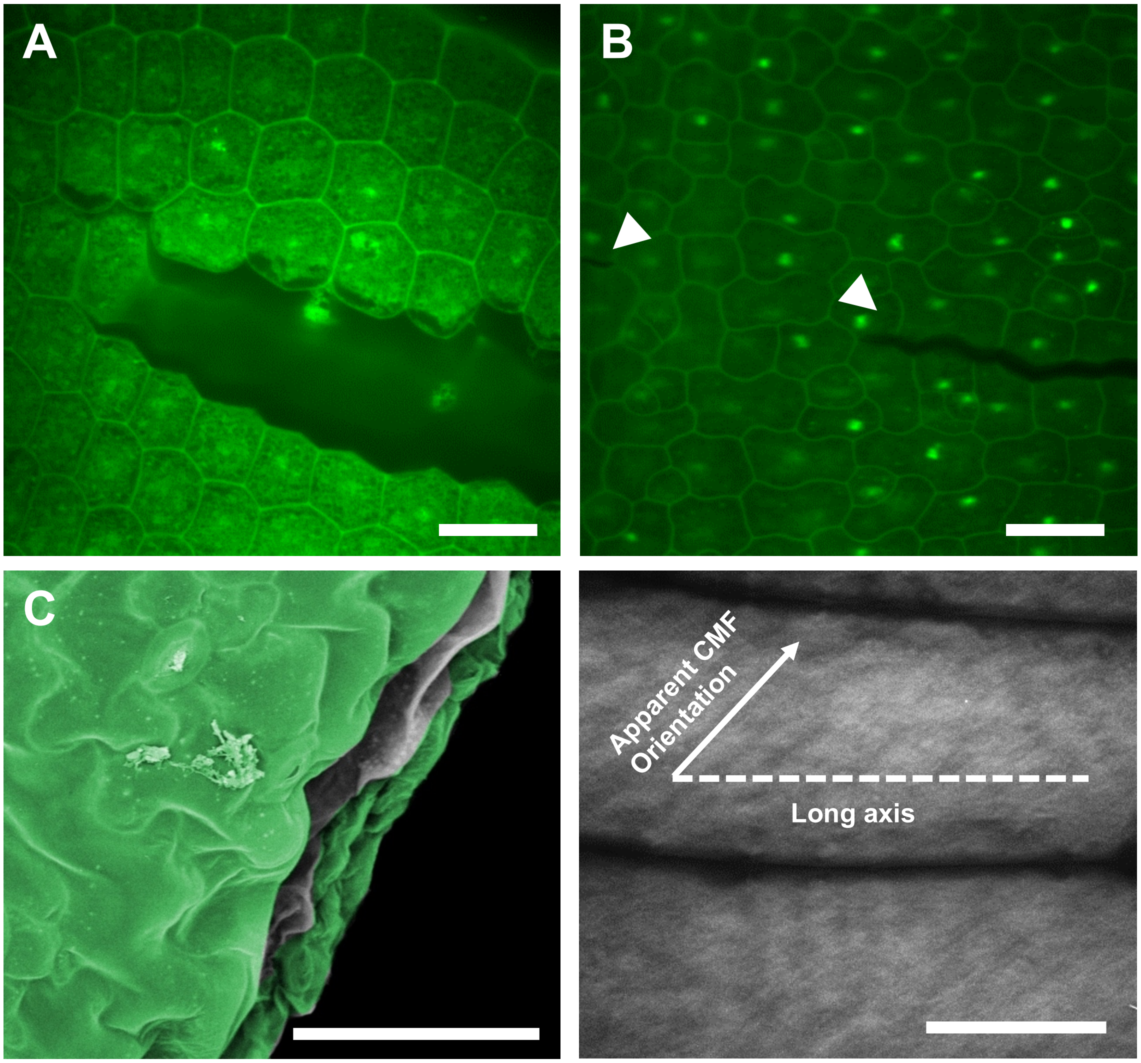

Supplementary Fig. 3. Fresh plant specimens. Confocal micrographs of a fresh *Arabidopsis* embryo stained with propidium iodide, squeezed between a glass slide and coverslip demonstrate cell-cell separation in **A**) radicle and **B**) cotyledon epidermal tissues. Micrograph is representative of at least three observations. **C)** A strip of fresh *Arabidopsis thaliana* leaf stretched to tear, then fixed and dehydrated for scanning electron microscopy. Green pseudocolor is used to mark the epidermis. Tear testing on fresh specimens was observed to produce artifacts preventing accurate detection of tear path due to warping of tear edges either after rupture or during sample preparation for scanning electron microscopy. To reveal fracture edges, subsequent fracture tests on multilayered leaves were performed on dehydrated samples instead. **D)** Apparent orientation of cellulose microfibrils in two adjacent adaxial onion epidermal cells stained with Pontamine Fast Scarlet 4B. Predominant orientation of cellulose microfibril bundles in onion epidermal cells can vary greatly, ranging from transverse to oblique to longitudinal directions depending on parameters such as location in scales and stage of cell growth. Micrograph is representative of at least three observations. Scale bars = 20 µm (A,B), 30 µm (C) and 50 µm (D).

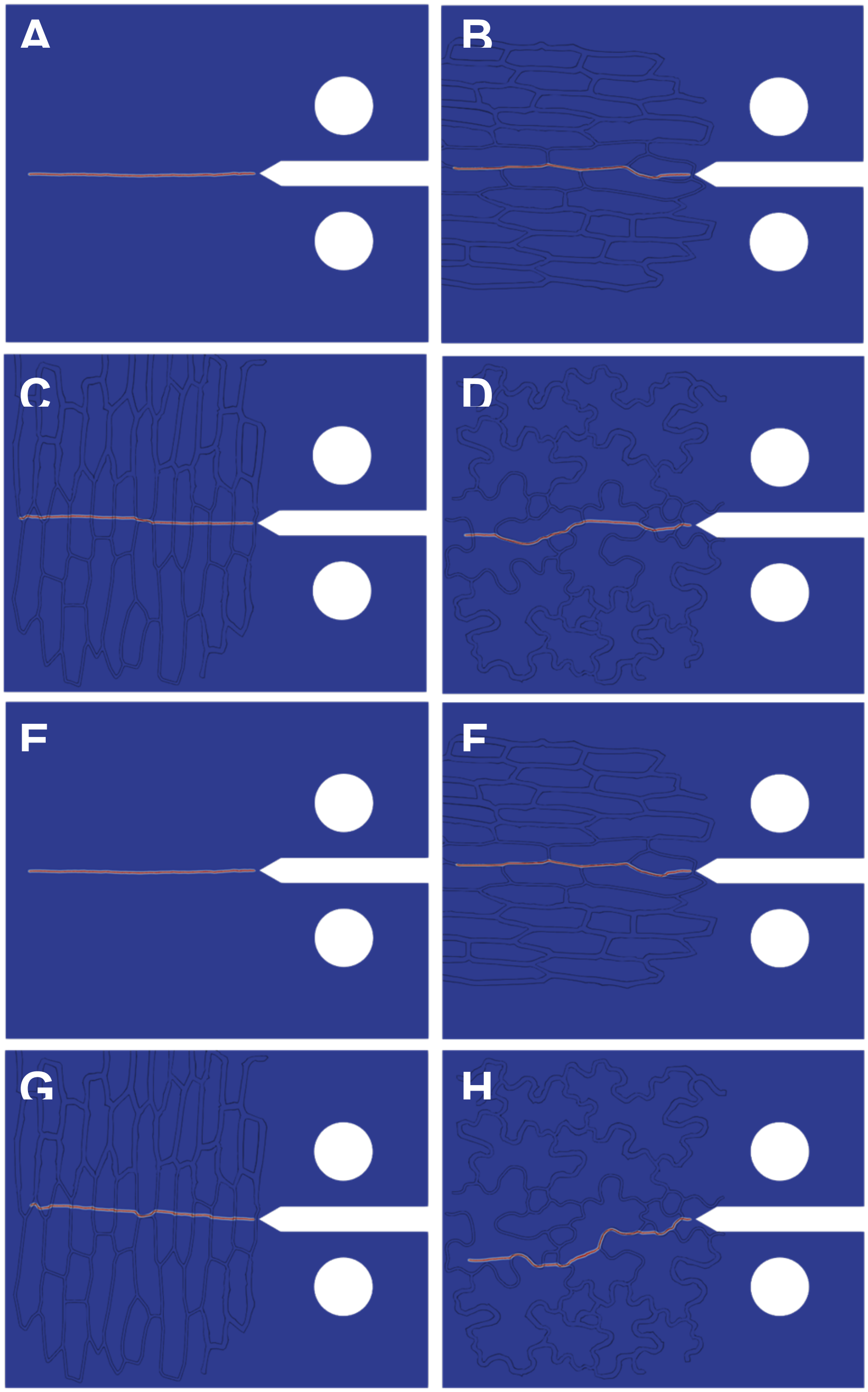

Supplementary Fig. 4. Phase-field fracture model crack propagation plots in original continuous distribution representation. A-D) Fracture behavior observed with the PFM for $E^{int} = E^{cell}$ and $G_c^{int} = G_c^{cell}/2$, and for **E-H)** $E^{int} = E^{cell}$ and $G_c^{int} = G_c^{cell}/2$.
