# Supplementary material for: Cell geometry regulates tissue fracture
Source: Nat Commun. 2023 Dec 13;14:8275. doi: 10.1038/s41467-023-44075-4 (PMC10719271; doi:10.1038/s41467-023-44075-4)
Supplement: Supplementary file 3 — Description of Additional Supplementary Files [file 41467_2023_44075_MOESM3_ESM.pdf]

Description of Additional Supplementary Files:

Supplementary Movie 1: Tear propagation parallel to cells in center-notched cellular tissue (onion epidermis). Upon initiation, tear propagates abruptly, reflected in the rapid drop of stretching force (8x speed). The graph demonstrates a real-time force (g) plot.

Supplementary Movie 2: Tear resistance perpendicular to cell alignment in center-notched cellular tissue (onion epidermis). Tear propagation demonstrating the resistance and reorientation of tear tips (8x speed), complemented by a real-time force (g) graph capturing the traversal resistance.

Supplementary Movie 3: Tear propagation parallel to cells in edge-notched cellular tissue (onion epidermis). Tear travels both in cell walls and in/adjacent to cell-cell interfaces (1x speed). In the latter case, tear propagates rapidly at decreased forces. The graph demonstrates a real-time force (g) plot.

Supplementary Movie 4: Tear resistance perpendicular to cell alignment in edge-notched cellular tissue (onion epidermis). Tear faces continued resistance as evident from the sustained force required for tearing which drops only slowly as tear propagates in the specimen (4x speed). The graph demonstrates a real-time force (g) plot.
